# Non-HLA genepolymorphisms in juvenile chronic arthritis: associations with outcome of disease

**DOI:** 10.1186/1546-0096-12-S1-P192

**Published:** 2014-09-17

**Authors:** Mikel Alberdi-Saugstrup, Lillemor Berntson, Christian Enevold, Anders Fasth, Susan M Nielsen, Ellen B Nordal, Marite Rygg, Marek S Zak, Klaus G Müller

**Affiliations:** 1 Children and Adolscent Clinic, Copenhagen University Hospital, Rigshospitalet, Copenhagen, Denmark; 2 Pediatric department, Naestved Hospital, Naestved, Denmark; 3 Department of Paediatrics, Uppsala University Hospital, Uppsala, Sweden; 4 Institute for Inflammation Research, Copenhagen University Hospital, Rigshospitalet, Copenhagen, Denmark; 5 Department of Pediatrics, University of Gothenburg, Gothenburg, Sweden; 6 Department of Pediatrics, University Hospital of North Norway, Tromsø, Norway; 7 Department of Laboratory Medicine, Children’s and Women’s Health, Norwegian University of Science and Technology, Norway; 8 Department of Pediatrics, St.Olavs Hospital, Trondheim, Norway

## Introduction

The etiology and pathogenesis of JIA are believed to be influenced by both genetic and environmental factors. Although several associations between the risk of developing JIA and gene variants both inside and outside the HLA region have been confirmed, no studies have examined associations between non-HLA gene polymorphisms and the disease outcome.

## Objectives

Genes involved in immune regulation and signal transduction of cytokines were studied. The genes chosen were previously shown to be associated with JIA in independent validation studies. They were selected to test the hypothesis that Single Nucleotide Polymorphisms (SNPs) previously shown to be associated with JIA are also risk factors for a severe disease outcome.

## Methods

A total of 500 children with JIA were included prospectively in a population based Nordic cohort study in 1997-2000. All patients had a recent diagnosis of JIA at inclusion. Not all centers were able to collect DNA from patients and at 8 year follow-up DNA was available from 217 patients. All eight categories of JIA were represented in the final cohort. Clinical data were collected longitudinally for the first eight years of disease. Remission was defined according to the preliminary criteria by Wallace et al. 2004. Testing of polymorphisms was performed using PCR based technology (KASPar v3.0 & v4.0 SNP Genotyping).

## Results

At the eight year follow-up visit 45% of the patients were in remission, on/off medication. Of the 11 SNPs examined, significant associations between genotypes and clinical endpoints were found for WISP3, IL2RA, STAT4 and VTCN1, although with low level of significance, p= 0.02-0.05 (Fisher’s exact test). Carriage of the A:A genotype of STAT4 was associated with persistently active disease eight years after disease onset (p=0.03) with about 7 times increased risk, but was not associated with onset type or the use of second line treatment (MTX and/or biologics). The other 7 tested SNPs were not associated with disease outcome.

## Conclusion

In a JIA cohort with longitudinally collected data, we were unable to show robust associations between selected non-HLA genotypes and disease outcome. Data were suggestive of associations between STAT4 A:A and an aggressive course of JIA in which remission were not achieved at eight year follow-up. This is of interest because STAT4 mediates the transcription of cytokine inducible genes and the A:A genotype has previously been associated with an increased risk of developing JIA. These findings should be confirmed in larger patient cohorts.

## Disclosure of interest

None declared.

**Table 1:** 

STAT4_rs3821236 genotype Vs. remission status			
	Remission (%)	No remission (%)	Total n.

A:A	1 (13)	7 (87)	8

G:A	37 (56)	30 (44)	67

G:G	47 (41)	68 (59)	115

Fisher's exact test	p = 0.03		

